# Identifying Life-Threatening Admissions for Drug Dependence or Abuse (ILIADDA): Derivation and Validation of a Model.

**DOI:** 10.1038/srep44428

**Published:** 2017-03-14

**Authors:** Tri-Long Nguyen, Thierry Boudemaghe, Géraldine Leguelinel-Blache, Céline Eiden, Jean-Marie Kinowski, Yannick Le Manach, Hélène Peyrière, Paul Landais

**Affiliations:** 1 Laboratory of Biostatistics, Epidemiology, Clinical Research and Health Economics, UPRES EA2415, University of Montpellier, Montpellier, France.; 2 Laboratory of Clinical Pharmacy, Faculty of Pharmacy, University of Montpellier, Montpellier, France.; 3 Department of Pharmacy, Nîmes University Hospital, Nîmes, France.; 4 Department of Biostatistics, Epidemiology, Public Health and Medical Informatics, Nîmes University Hospital, Nîmes, France.; 5 Department of Medical Pharmacology and Toxicology, Addictovigilance Centre, Montpellier University Hospital, Montpellier University Hospital, Montpellier, France.; 6 Departments of Anesthesia & Clinical Epidemiology and Biostatistics, Michael DeGroote School of Medicine, Faculty of Health Sciences, McMaster University, Hamilton, Ontario, Canada.; 7 The Perioperative Research Group, Population Health Research Institute, Hamilton, Ontario, Canada.

## Abstract

Given that drug abuse and dependence are common reasons for hospitalization, we aimed to derive and validate a model allowing early identification of life-threatening hospital admissions for drug dependence or abuse. Using the French National Hospital Discharge Data Base, we extracted 66,101 acute inpatient stays for substance abuse, dependence, mental disorders or poisoning associated with medicines or illicit drugs intake, recorded between January 1^st^, 2009 and December 31^st^, 2014. We split our study cohort at the center level to create a derivation cohort and a validation cohort. We developed a multivariate logistic model including patient’s age, sex, entrance mode and diagnosis as predictors of a composite primary outcome of in-hospital death or ICU admission. A total of 2,747 (4.2%) patients died or were admitted to ICU. The risk of death or ICU admission was mainly associated with the consumption of opioids, followed by cocaine and other narcotics. Particularly, methadone poisoning was associated with a substantial risk (OR: 35.70, 95% CI [26.94–47.32], P < 0.001). In the validation cohort, our model achieved good predictive properties in terms of calibration and discrimination (c-statistic: 0.847). This allows an accurate identification of life-threatening admissions in drug users to support an early and appropriate management.

Worldwide, illicit drug use and dependence are important contributors to the global burden of disease, accounting for 20 million disability-adjusted life years^1^. This burden is mainly associated with opioid dependence and increases in highest incomes countries^1^. In the United States, drug overdose deaths have increased significantly recent years^2^. A wide variety of healthcare, social and educational settings are involved in the identification and treatment of substance abuse- and dependence-related disorders^3^. Admissions to acute care hospitals for drug abuse or dependence are common and likely associated with complications.

Recently, it has been suggested that drug users were at higher risk for intensive care unit (ICU) admission than the general population^4^. Life-threatening hospital admissions need an early and appropriate management, and sometimes require supportive care^5^,^6^,^7^. In drug-overdosed patients, the immediate need for ICU monitoring can be assessed with tools such as the Glasgow coma score^8^. However, the latency of certain toxic syndromes can bias the initial evaluation and thus lead to overestimating the prognosis. Particularly, a secondary deterioration could be observed in patients initially admitted for behavioral disorders due to an acute intoxication, if no appropriate and early management is initiated. Accurately identifying potential life-threatening cases at admission is therefore of utmost importance and it could result in the respiratory and hemodynamic stabilization of the patient before ICU admission^6^. We propose to provide early prognostic information based on patient characteristics at hospital entrance, including the suspected responsible agent. To the best of our knowledge, such an approach has not been previously described in the medical literature for identifying life-threatening cases in drug users admitted to acute care hospitals.

In this study, we report both the derivation and the validation of a model allowing an early identification of life-threatening admissions for drug dependence or abuse (ILIADDA).

## Methods

### Ethics statement

This observational study was conducted according to the authorization given by the *Agence pour le Traitement de l’Information Médicale* (ATIH), which waived the need for patient’s consent according to the Enforcement (decree No. 94-666). All methods were carried out in accordance with relevant guidelines on prognosis study and the protocol was approved by the *Commission Nationale de l’Informatique et des Libertés* (agreement No. 1375062), as required by the French Protection Act. The data provided were de-identified.

### Data source and study population

In France, the National Hospital Discharge Data Base (NHDDB) records complete anonymized information on all patients, in all French private and public hospitals. It provides discharge summaries of acute hospitalizations, which are grouped into Diagnosis-Related Groups (DRGs)^9^. For each DRG, an annual tariff is calculated at the national level based on average treatment costs.

We extracted all of the discharge summaries, recorded within the NHDDB from January 1^st^, 2009 to December 31^st^, 2014, reporting a DRG for substance abuse, dependence, mental disorders or poisoning associated with medicines or illicit drugs intake (see Supplemental Digital Content for the DRG codes). Admissions related to tobacco or alcohol disorders were not included.

### Outcomes and predictors

We defined the primary endpoint as a composite outcome of ICU admission or in-hospital death.

In order to allow the clinician to predict the patient’s risk for the occurrence of the primary outcome at hospital admission, we considered the potential predictors as those available at entrance (time origin): age, sex, entrance by transfer and the diagnosis that motivated the hospitalization. This diagnosis at admission was recorded according to the International Classification of Diseases, 10^th^ Revision (ICD-10)^10^.

### Statistical analysis

For descriptive purposes, means (standard deviations, SD) and frequencies (percentages) were reported for continuous and categorical variables, respectively. In addition, we reported the temporal distribution of acute admissions related to drug dependence or abuse and we mapped the geographical distribution. The number of admissions by location was standardized on the number of inhabitants reported by the *Institut National de la Statistique et des Études Économiques*^11^. The map was created with © Géoclip 2015 – IGN GéoFla.

For predictive purposes, implementing a prognostic model in clinical practice routine must be preceded by three steps: the model derivation, the model validation and the clinical impact assessment^12^,^13^,^14^,^15^,^16^,^17^,^18^,^19^,^20^,^21^. In this study, we reported both the derivation and the validation of a model, according to the TRIPOD statement^12^,^13^. Given that the predictive performance of a model is likely to be optimistic relative to the data used for the model derivation, it must be evaluated on an independent set of patients. To this end, we split our study cohort into a derivation cohort and a validation cohort. There is a clear consensus as to the inefficiency of randomly splitting a cohort at the patient level, because it creates two very similar cohorts that only differ by chance^12^,^13^,^22^,^23^,^24^. Splitting by time (temporal or “narrow” validation) or by location (geographic or “broad” validation) is a more reliable approach^12^,^13^. To conduct a broad validation, we split the entire cohort at the center level (ratio 2:1 for derivation and validation, respectively). This allowed us to assess the performance of our model in centers, within which practices and measurements were likely to differ from those of the derivation cohort^25^.

In the derivation cohort, we performed a multivariate logistic regression (maximum likelihood estimation) adjusting the risk of death or ICU admission for several predictors. Due to the large sample size, selecting predictors according to the P-value would have led to a non-parsimonious model. Therefore, we limited the set of binary predictors to those for which 10 events (or more) occurred. For the continuous variable, we used a restricted cubic spline with three knots to handle the non-linearity of age effect. The efficiency of this procedure has been previously demonstrated for prediction modeling^26^. To provide a parsimonious and clinically usable final model, we removed the predictors associated with less than 0.25% of the multivariate model deviance, without damaging the predictive performance in terms of discrimination and calibration. Discrimination refers to the ability of the model to separate patients with different outcomes (*i.e.* those with life-threatening events *versus* those without), while calibration refers to the agreement between observed and predicted outcomes. In all analyses, multiple admissions for the same patient were considered as independent stays. Indeed, including the number of previous admissions per patient did not improve the model performance, while left-truncation dramatically reduced the sample size. In both the derivation cohort and the validation cohort, we reported the model discrimination (c-statistic: the area under the curve plotting *sensitivity* against *1 − specificity*), overall accuracy (Brier score) and calibration (logistic regression of the observed outcome on the predictions for estimating the slope and intercept, and local weighted regression curve with span = 0.75 for graphical assessment). Confidence intervals of performance metrics were calculated by bootstrapping (500 iterations). All statistical analyses were carried out in R software (version 3.3).

### Patient and funding organization involvement

No patients were involved in setting the research question; nor were they involved in the design of the study or the outcome measures. No funding organizations were involved in the study.

## Results

### Acute hospitalization in drug users

**Figure 1:**
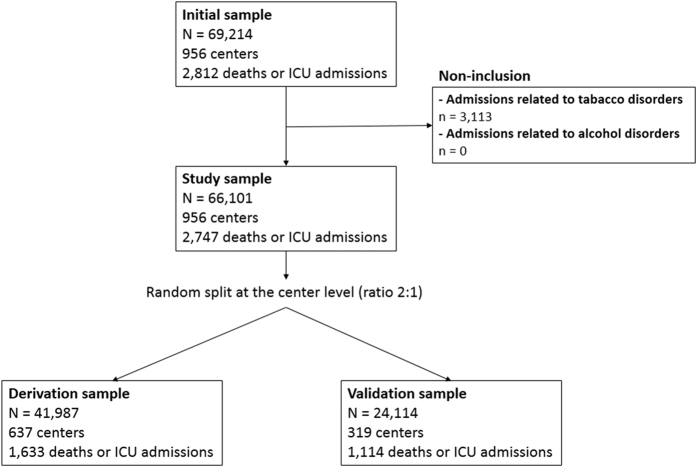
Admissions flowchart.

**Figure 2:**
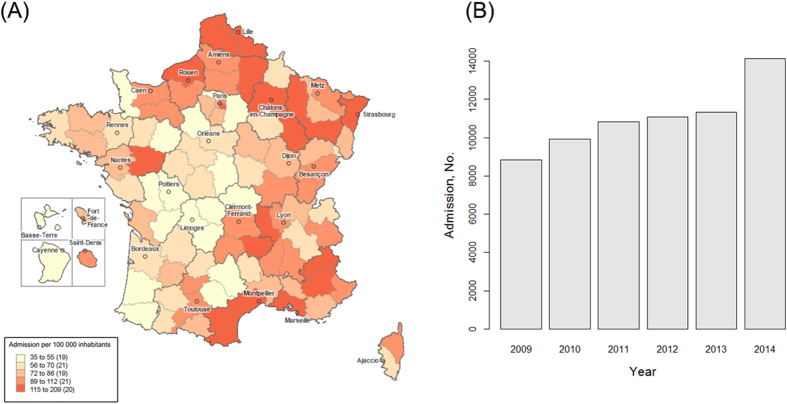
Admission to acute care hospitals for drug abuse or dependence in France, according to the National Hospital Discharge Data Base (2009–2014). The map was constructed with © Géoclip 2015 – IGN GéoFla (http://franceo3.geoclip.fr/#v=map6;l=fr;z=-1132292,6721213,2779367,1730915).

From January 2009 to December 2014, we reported a total of 66,101 acute admissions for drug abuse or dependence in 956 centers (Fig. 1). During this period, there was a substantial increase in the number of hospital admissions (from 8,834 admissions in 2009 to 14,118 admissions in 2014, +59.8%), which was associated with a widespread geographical distribution (Fig. 2). The mean age was 37.4 years (SD: 16.8) and 39,325 patients (59.5%) were male. Nine hundred and ninety-five patients (1.5%) entered by transfer from another structure. The mean length of stay was 3.6 days (SD: 5.7). A large majority of these admissions (61,875, 93.4%) occurred in public hospitals with a mean tariff of €1,704 (SD: 1,895).

As summarized in Table 1, the main reason for admission was drug rehabilitation (19,319 admissions, 29.2%), including rehabilitation after drug abuse, drug detoxification, drug replacement therapy, drug withdrawal and treatment of withdrawal symptoms. Others were mostly related to the consumption of opioids (15,951 admissions, 24.1%), sedative or hypnotics (8,973 admissions, 13.6%), cannabinoids (7,445 admissions, 11.3%) and cocaine (2,709 admissions, 4.1%).

### Risk of ICU admission or in-hospital death

In the entire cohort, the primary outcome occurred in 2,747 stays (4.2%), with 2,602 admissions to ICU and 211 in-hospital deaths. Of these deaths, 66 occurred in ICU (mortality rate in ICU: 2.5%). The mean SAPS II score at admission to ICU was equal to 35.0 (SD: 17.4).

For risk modeling, we split the study cohort at the hospital center level: 41,987 admissions in 637 centers were used for the model derivation, and 24,114 admissions to 319 centers for the model validation. A total of 1,633 (3.9%) and 1,114 (4.6%) deaths or ICU admissions occurred in the derivation cohort and in the validation cohort, respectively.

We reported a multivariate logistic model including 17 predictors (Table 2). Poisoning and acute intoxication by opioids were associated with a substantial increased risk of death or ICU admission: methadone poisoning OR: 35.70 (95% CI [26.94–47.32], P < 0.001), heroin poisoning OR: 22.72 (95% CI [15.84–32.61], P < 0.001), opium poisoning OR: 18.26 (95% CI [10.45–31.89], P < 0.001), poisoning by other opioids OR: 20.17 (95% CI [15.69–25.92], P < 0.001) and behavioral disorders due to an acute opioids intoxication OR: 17.61 (95% CI [13.49–22.97], P < 0.001). Poisoning and acute intoxication by narcotics other than opioids also increased the outcome risk: cocaine poisoning OR: 12.70 (95% CI [8.09–19.93], P < 0.001), behavioral disorders due to an acute cocaine intoxication OR: 3.12 (95% CI [1.69–5.79], P < 0.001), poisoning by other synthetic narcotics OR: 8.99 (95% CI [6.26–12.91], P < 0.001) and poisoning by other and unspecified narcotics OR: 18.97 (95% CI [13.26–27.14], P < 0.001). Other drugs had a lower, but still substantial effect on the risk of death or ICU admission (Table 2). In contrast, rehabilitation of drug users was associated with a decreased risk of occurrence of the primary outcome, OR: 0.18 (95% CI [0.11–0.29], P < 0.001), as well as the female sex, OR: 0.86 (95% CI [0.77–0.96], P < 0.001). The age effect followed an “n-shaped” curve with a maximal risk at 44 years old, OR: 7.98 (Supplemental Digital Content).

The model can be used to predict the individual risk of a life-threatening event with the formula:


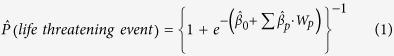


For each individual, *W*_*p*_ denotes his/her value for the *p*^*th*^ predictor, with 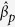 the corresponding estimated regression coefficient (*i.e.* logarithm of the OR of the *p*^*th*^ predictor) and 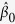 the estimated constant as reported in Table 2.

**Figure 3:**
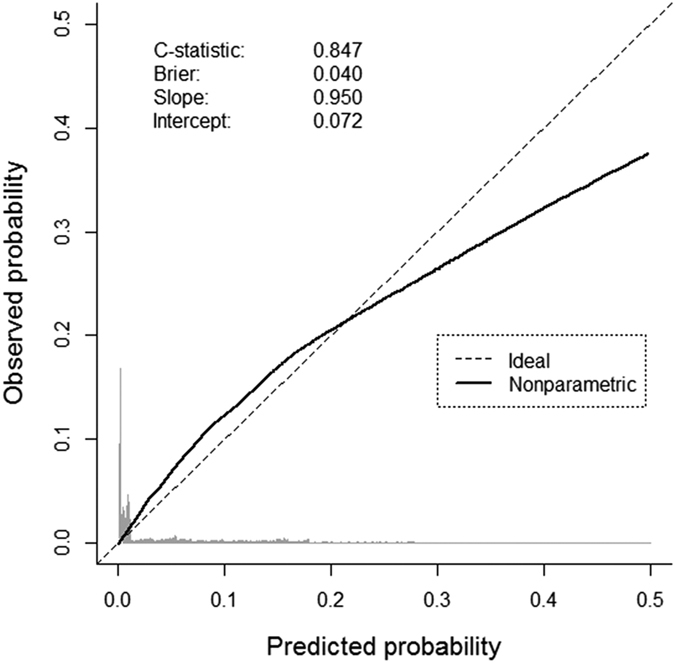
Calibration plot comparing predicted *versus* observed deaths or ICU admissions in the validation sample.

In the derivation cohort, our model achieved good predictive performances: c-statistic 0.851 (95% CI [0.844–0.859]) and Brier score 0.034 (95% CI [0.033–0.036]). This was confirmed in the validation cohort: c-statistic 0.847 (95% CI [0.838–0.855]) and Brier score 0.040 (95% CI [0.038–0.043]). The overall calibration slope in the validation cohort (0.950) depicted a slight over-fitting issue of the model development. Using a local weighted regression curve, we compared the predicted probabilities with the observed outcome frequencies (Fig. 3). In the validation cohort, we reported a small overestimation of the predicted probabilities beyond 0.2, which only concerned a few patients.

## Discussion

We report the derivation and validation of a model, which demonstrates an accurate prediction of death or ICU admission in hospitalized drug users.

To the best of our knowledge, we provide the first model allowing an early identification of life-threatening cases in drug users admitted to acute care hospitals. This is of great importance as, in contrast with diagnosis scores (e.g., Glasgow coma score) that assess the immediate need for intensive care and are likely to vary according to the latency of the “toxidrome”, our model provides accurate prognostic information at hospital entrance on the risk of life-threatening events. In response to this clinical issue, our model includes only predictors that are readily available at hospital admission. Though we recognize that comorbidities, cognitive status, medications and biological markers are potential predictors, we did not take them into account in our model since these are not systematically investigated in every patient upon admittance. Had we considered those potential predictors would have led to limiting the usability of our model only to patients whose comorbidities, cognitive status, medications and biological markers are recorded at admission. Indeed, in the case of missing values regarding one of those predictors (*i.e.* absence of input while using the corresponding model formula) the predicted probability of life-threatening event would no longer be calculable. Without including those variables, our model still demonstrates good predictive properties in terms of discrimination and calibration. This might therefore be a useful tool for routine practice, supporting clinical decision-making to improve the early management of critically ill patients, who increasingly contribute to the global burden of healthcare.

Drug abuse or dependence is a common reason for admission into acute care hospitals. In our large French nationwide cohort, we found that 4.2% of these hospitalizations led to death or ICU admission. This is concordant with the 3% of empoisoned patients requiring critical care in the United States^7^. Although these life-threatening cases are limited, they require an early and appropriate management^5^,^6^,^27^, which motivated the development and validation of our model. Predictors include early diagnosis, which is commonly revealed by specific clinical syndromes (“toxidromes”) suggesting particular substances. Life-threatening cases typically correspond to male adults within the range of 30–50 years old and involving an acute intoxication or poisoning by opioids. A rapid identification is essential, as patient’s respiratory and hemodynamic stabilization can be achieved before ICU admission^6^. As previously reported in other Western countries^28^,^29^, methadone poisoning corresponds to the greatest risk, followed by the other opioids. Indeed, in France, the nationwide DRAMES study (*Décès en Relation avec l’Abus de Médicaments et de Substances –* Death Related to Medication and Substance Abuse) identified methadone as the main drug responsible for death, above heroin and cocaine^30^,^31^. This high risk associated to methadone poisoning might be related to its long duration of action. Hall and colleagues showed that drug overdose deaths were mostly associated with diversion of opioid analgesics, particularly methadone^29^. To address the problem of prescription drug abuse, the American College of Physicians has recently published a position paper that provides guidance to prescribers and policymakers^32^. According to recent American studies, there has been a downturn trend in opioid analgesics abuse within the decade, which was associated with an increased shift to heroin abuse in a more widespread geographical distribution^33^,^34^,^35^,^36^. In terms of prognosis, our data suggest that the risk of death or ICU admission associated with heroin poisoning is lower than with methadone poisoning, but higher than other opioids.

Following opioids, cocaine and other narcotics are the next most responsible for life-threatening admissions. The risk of death or ICU admission related to cocaine poisoning is about half of that associated with methadone, but is still substantial^37^. Other drugs responsible for life-threatening admissions are hallucinogens, sedatives or hypnotics, and cannabis or derivatives. In contrast to acute poisoning or intoxication, drug rehabilitation is associated with a reduced risk of outcome. Further studies are needed to demonstrate whether chronic care management is efficient^38^.

One of the strengths of our study is using a complete nationwide database. The large sample size enabled a broad model validation. It assessed the performances of our model on centers that were not used for the model derivation. All of these analyses were reported with transparency.

Our study has to be considered in the context of its limitations. First, the lack of sensitivity of the French medico-administrative database for identifying drug-dependent patients has been previously discussed^39^. However, this issue has been highlighted in the context of a routine detection of drug users. We recognize that the French NHDDB should not be used for drug user detection, as the population admitted to acute care hospitals does not reflect the overall drug-addicted population, who is likely to benefit from a wide variety of non-healthcare, social and educational settings. However, such a lack of sensitivity did not concern our study, since we focused on the most severe acute admissions, which were specifically motivated by substance abuse, dependence, mental disorders or poisoning associated with medicines or illicit drug intake. Use of this database should not give rise to an excessive number of false-positive cases, as the French NHDDB is primarily used for reimbursement purposes and is unlikely inclined to misdiagnoses^39^. This database records for each stay the diagnosis that resulted in hospitalization and the associated comorbidity (when applicable), which follow the International Classification of Diseases, 10^th^ Revision. The granularity of this classification does not enable to describe the precise clinical spectrum of the disease and its evolution. Though medico-administrative databases can be regarded as relevant tools for describing admissions specifically related to illicit drugs consummation^40^, their potential use for clinical toxicology is still limited^39^,^41^,^42^. In certain situations, the information on drugs responsible for admission were limited to the drug classes stated by the ICD-10, which did not allow us to explore the risk related to some specific drugs, in particular, prescription opioids (e.g. oxycodone). Moreover, the history of drug abuse is not collected as such in the database, which is a limitation of our approach. Additionally, predictors such as individual socio-demographic characteristics were not available in our database. In spite of these limits, our model demonstrated good predictive performances. Nevertheless, the effect of its implementation on the decision-making process has not been measured, thus a further study is needed to assess its clinical impact, before implementation in routine clinical practice.

## Conclusions

We report a simple model, which predicts the risk of death or ICU admission in drug users with good discriminative and calibration properties. This model, which includes patients’ characteristics at hospital admission might be useful in clinical practice for early identification of life-threatening cases.

## Additional Information

**How to cite this article**: Nguyen, T.-L. *et al*. Identifying Life-Threatening Admissions for Drug Dependence or Abuse (ILIADDA): Derivation and Validation of a Model. *Sci. Rep.* **7**, 44428; doi: 10.1038/srep44428 (2017).

**Publisher's note:** Springer Nature remains neutral with regard to jurisdictional claims in published maps and institutional affiliations.

## Supplementary Material

Supplementary material

## Figures and Tables

**Table 1:** Primary diagnoses at admission.

Diagnosis (ICD-10 code)	No. (%)
Drug rehabilitation (Z503)	19,319 (29.2%)
*Opioids*	*15,951 (24.1%)*
Poisoning by opium (T400)	214 (0.3%)
Poisoning by heroin (T401)	548 (0.8%)
Poisoning by other opioids (T402)	6,953 (10.5%)
Poisoning by methadone (T403)	1,358 (2.1%)
Mental or behavioral disorders due to use of opioids (F11)	6,871 (10.4%)
Finding of opiate drug in blood (R781)	7 (0.0%)
Mental or behavioral disorders due to the use of sedatives or hypnotics (F13)	8,973 (13.6%)
*Cannabinoids*	*7,445 (11.3%)*
Poisoning by cannabis (derivatives) (T407)	1,694 (2.6%)
Mental or behavioral disorders due to the use of cannabinoids (F12)	5,751 (8.7%)
*Cocaine*	*2,709 (4.1%)*
Poisoning by cocaine (T405)	553 (0.8%)
Mental or behavioral disorders due to use of cocaine (F14)	2,147 (3.2%)
Finding of cocaine in blood (R782)	9 (0.0%)
Poisoning by other synthetic narcotics (T404)	1,519 (2.3%)
Poisoning by other and unspecified narcotics (T406)	1,072 (1.6%)
Poisoning by lysergide [LSD] (T408)	110 (0.2%)
Poisoning by other and unspecified psychodysleptics (T409)	434 (0.7%)
Mental or behavioral disorders due to the use of hallucinogens (F15)	393 (0.6%)
Mental or behavioral disorders due to the use of other stimulants (F16)	415 (0.6%)
Mental or behavioral disorders due to the use of volatile solvents (F18)	176 (0.3%)
Mental or behavioral disorders due to multiple drug use and use of psychoactive substances (F19)	5,785 (8.8%)
Others	1,800 (2.7%)

**Table 2:** Model for identifying life-threatening admissions for drug dependence or abuse (ILIADDA).

	Regression coefficient	Odds ratio [95% CI]	*P-value*
*Constant*	−6.615		
Age (y)	0.060	1.06 [1.05–1.07]	<0.001
(Age)′	−0.061	0.94 [0.93–0.95]	<0.001
Female sex	−0.155	0.86 [0.77–0.96]	0.006
Transfer at entrance	0.957	2.60 [1.99–3.41]	<0.001
*Reason for hospital admission (ICD-10 code*)			
Drug rehabilitation (Z503)	−1.712	0.18 [0.11–0.29]	<0.001
*Opioids*			
Poisoning by methadone (T403)	3.575	35.70 [26.94–47.32]	<0.001
Poisoning by heroin (T401)	3.123	22.72 [15.84–32.61]	<0.001
Poisoning by opium (T400)	2.905	18.26 [10.45–31.89]	<0.001
Poisoning by other opioids (T402)	3.004	20.17 [15.69–25.92]	<0.001
Mental and behavioral disorders due to use of opioids, acute intoxication (F110)	2.868	17.61 [13.49–22.97]	<0.001
*Cocaine and other narcotics*			
Poisoning by cocaine (T405)	2.542	12.70 [8.09–19.93]	<0.001
Mental and behavioral disorders due to use of cocaine, acute intoxication (F140)	1.139	3.12 [1.69–5.79]	<0.001
Poisoning by other synthetic narcotics (T404)	2.196	8.99 [6.26–12.91]	<0.001
Poisoning by other and unspecified narcotics (T406)	2.943	18.97 [13.26–27.14]	<0.001
*Others*			
Poisoning by cannabis (derivatives) (T407)	1.207	3.34 [1.93–5.78]	<0.001
Poisoning by other and unspecified psychodysleptics [hallucinogens] (T409)	2.105	8.21 [4.71–14.30]	<0.001
Mental and behavioral disorders due to use of sedatives or hypnotics, acute intoxication (F130)	1.817	6.16 [4.73–8.02]	<0.001
Mental and behavioral disorders due to multiple drug use and use of psychoactive substances, acute intoxication (F190)	1.908	6.74 [4.89–9.28]	<0.001

A restricted cubic spline with three knots was used to describe the effects of age (knots at 19, 36 and 59 years).
