# Supplementary material for: Identifying Life-Threatening Admissions for Drug Dependence or Abuse (ILIADDA): Derivation and Validation of a Model
Source: Sci Rep. 2017 Mar 14;7:44428. doi: 10.1038/srep44428 (PMC5349588; doi:10.1038/srep44428)

**Identifying Life-Threatening Admissions for Drug Dependence or Abuse (ILIADDA): Derivation and Validation of a Model.**

Tri-Long Nguyen, Pharm.D. Ph.D., Thierry Boudemaghe, M.D. M.Sc., Géraldine Leguelinel-Blache, Pharm.D. Ph.D., Céline Eiden, Pharm.D. Ph.D., Jean-Marie Kinowski, Pharm.D. Ph.D., Yannick Le Manach, M.D. Ph.D., Hélène Peyrière, Pharm.D. Ph.D., Paul Landais, M.D. Ph.D.

# *Supplemental Digital Content*

**Table S1.** Inclusion criteria. The reliability index reflects the data consistency (5 as maximal value). Remaining admissions with a main diagnosis of tobacco- or alcohol-related disorders were not included in the study sample.

| **French diagnosis-related group** | **Code** | **Main diagnosis (ICD-10 code)** | **Reliability index** |
| --- | --- | --- | --- |
| Mental or behavioral disorders induced by alcohol or other substances | 20Z06V | F10-, F11-, F12-, F13-, F14-, F15-, F16-, F18-, F19- | 5 |
| Intoxication by drugs or biological substances, < 18 years old | 21M02 | T40- | 5 |
| Intoxication by drugs or biological substances, > 17 years old | 21M10 | T40- | 5 |
| Non-alcoholic drug addiction, with dependence | 20Z02 | No restriction | 5 |
| Non-alcoholic drug abuse, without dependence | 20Z03 | No restriction | 5 |

**Figure S1.** The effect of age on the risk of death or ICU admission in drug users. A restricted cubic spline with three knots was used to handle the non-linearity (knots at 19, 36 and 59 years).


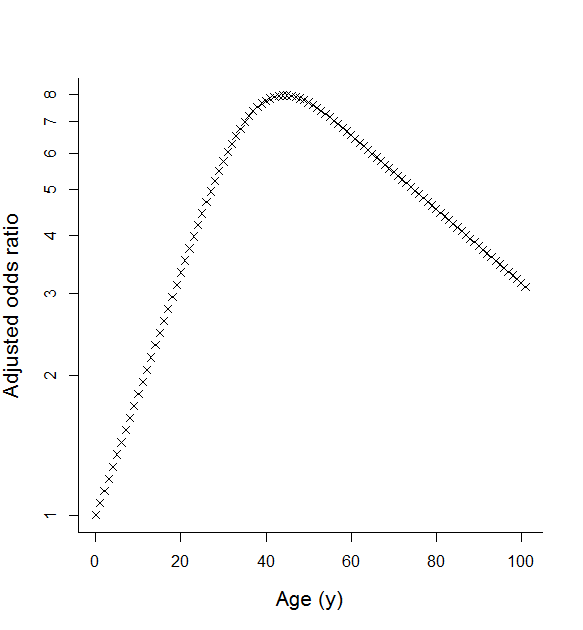

Supplement: Supplementary material [file srep44428-s1.doc]
